# Supplementary material for: MIR17HG Expression Is Transcriptionally Regulated by PAX3::FOXO1 and MYCN and is Necessary for Oncogenic Activity in Fusion-Positive Rhabdomyosarcoma
Source: bioRxiv. 2025 Nov 22:2025.11.21.689335. Preprint. [Version 1] doi: 10.1101/2025.11.21.689335 (PMC12747281; doi:10.1101/2025.11.21.689335)
Supplement: Supplement 2 [file NIHPP2025.11.21.689335v1-supplement-2.pdf]

Supplementary Figures

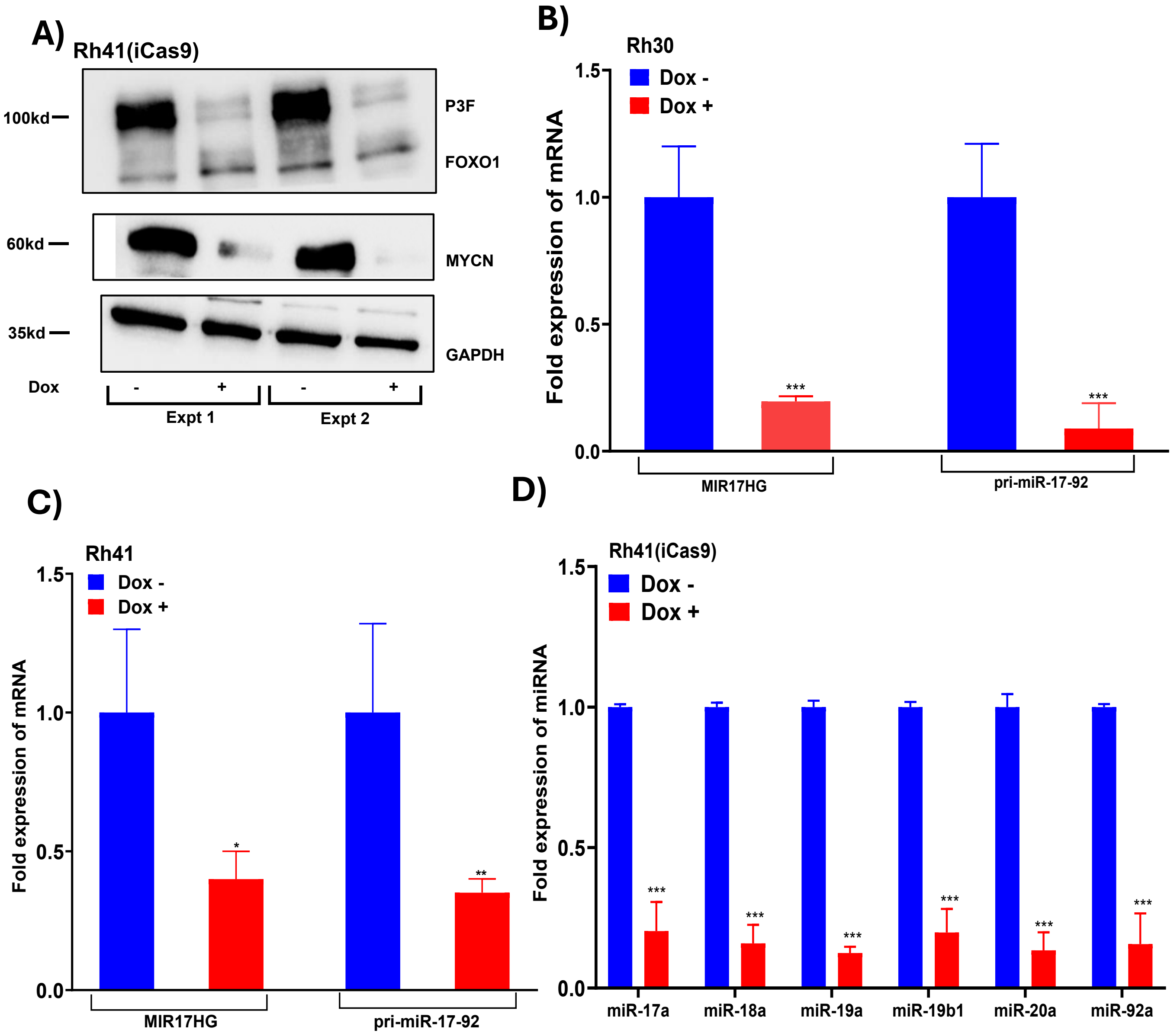

Figure S1. CRISPR-Cas9 knockout of P3F suppresses *MIR17HG* transcript, pri-miR-17-92 and mature miRNAs in FP-RMS cells. A. Western blot of P3F and MYCN protein in Rh41 cells treated without (-) or with (+) 2000 ng/ml doxycycline (Dox) to express Cas9. Two independent experiments are shown. **B, C.** qPCR analysis of *MIR17HG* transcript and pri-miR-17-92 in Rh30 (B) and Rh41 (C) following Dox-induced knockout of *P3F* in the Cas9-inducible subclones. **D.** qPCR analysis of mature miRNAs within the miR-17-92 cluster following Dox-induced knockout of *P3F* in the Cas9-inducible Rh41 cells. In B, C and D, cells were treated without (-) and with (+) Dox, and results are normalized for cells without Dox (-). Statistical significance is displayed as: not significant (ns), $p < 0.05$ (*), $p < 0.01$ (**), $p < 0.001$ (***).

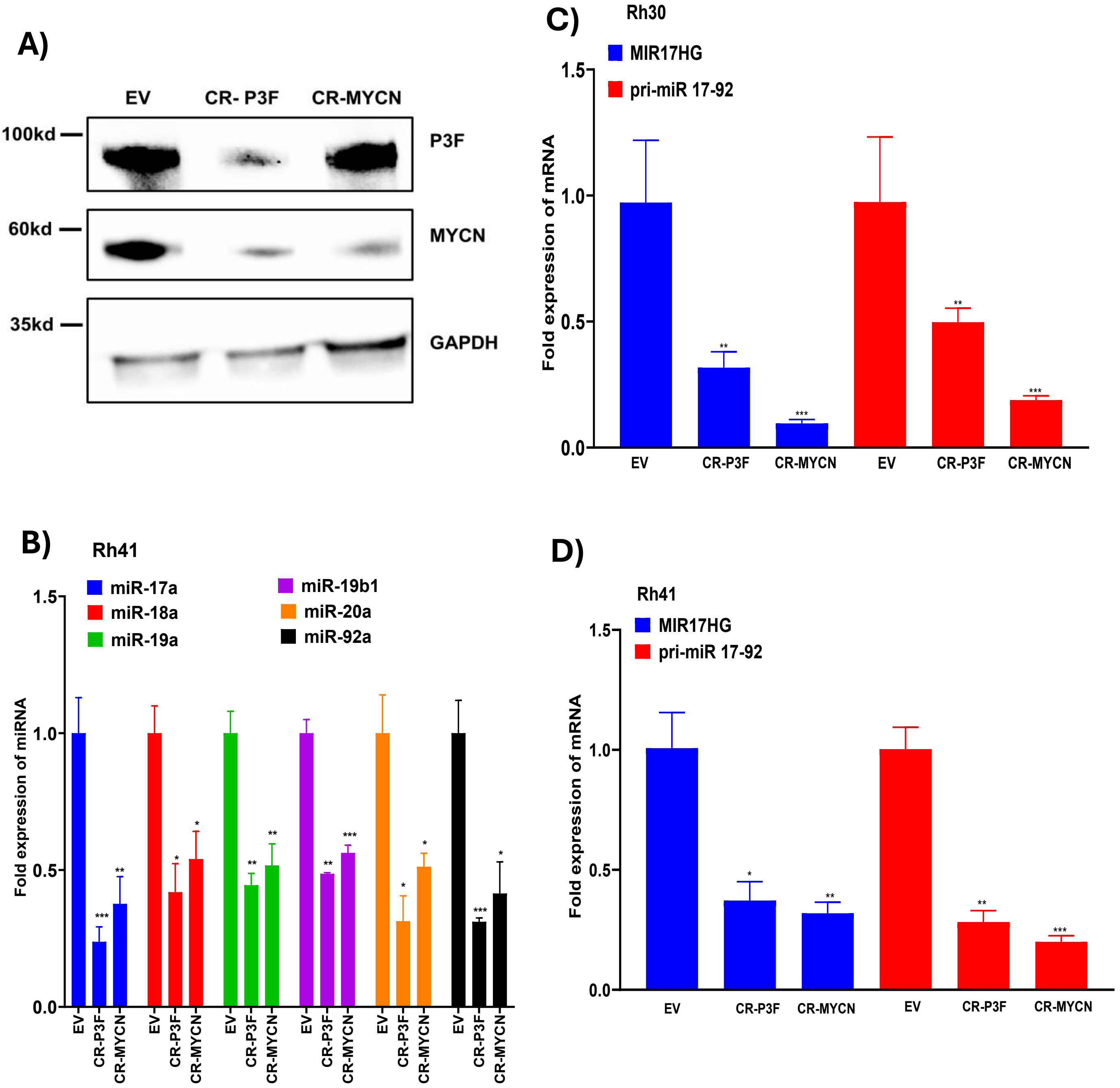

Figure S2. CRISPR-Cas9 knockout of P3F or MYCN suppresses MIR17HG transcript, pri-miR-17-92 and miR-17-92 cluster expression in FP-RMS cells. A, B. Western blot analysis of P3F and MYCN (A) and qPCR analysis of expression of mature miRNAs from the miR-17-92 cluster (B) following CRISPR-Cas9-mediated knockout (CR) of *P3F* or *MYCN* in Rh41 cells. Control cells were transduced with empty vector (EV). **C, D.** qPCR analysis of expression of *MIR17HG* transcript and pri-miR-17-92 following CRISPR-Cas9-mediated knockout of *P3F* or *MYCN* in Rh30 (C) and Rh41 (D) cells. In B, C, and D, results are normalized for control cells and are presented as mean ± SD from at least three independent experiments. Statistical significance is displayed as described in Figure S1.

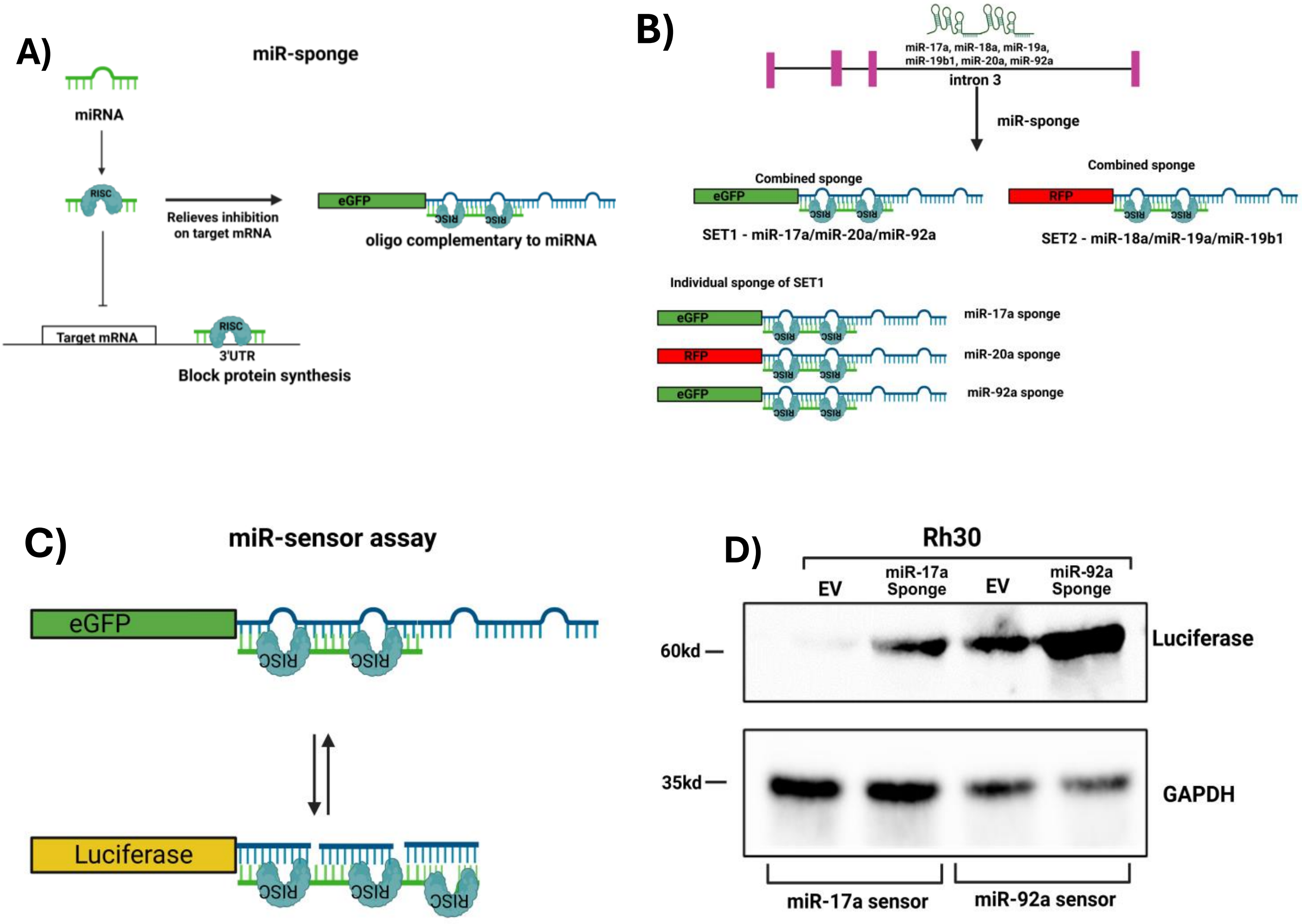

Figure S3. Inhibition of miR-17-92 with miRNA-sponges. A. Schematic representation of miRNA-sponge mechanism, illustrating synthetic constructs with tandem binding sites partially complementary to miRNA designed to sequester endogenous miRNAs and relieve repression of target mRNAs. **B**. Schematic of miRNA sponge design for the miR-17-92 cluster. The six miRNAs encoded within *MIR17HG* intron 3 were divided into two sponge constructs: SET1 (eGFP; miR-17a/miR-20a/miR-92a) and SET2 (RFP; miR-18a/miR-19a/miR-19b1). SET1 was further separated into individual sponges targeting miR-17a (eGFP), miR-20a (RFP), and miR-92a (eGFP). **C**. Schematic of the miRNA sensor assay. Sensor vectors contain three tandem binding sites with perfect complementarity to miR-17a or miR-92a placed in the 3' UTR of a luciferase reporter gene. **D**. Western blot analysis of luciferase protein in doxycycline-treated Rh30 cells transduced with miR-17a sensor with EV or miR-17a sponge, or miR-92a sensor with EV or miR-92a sponge. GAPDH was utilized as a loading control.

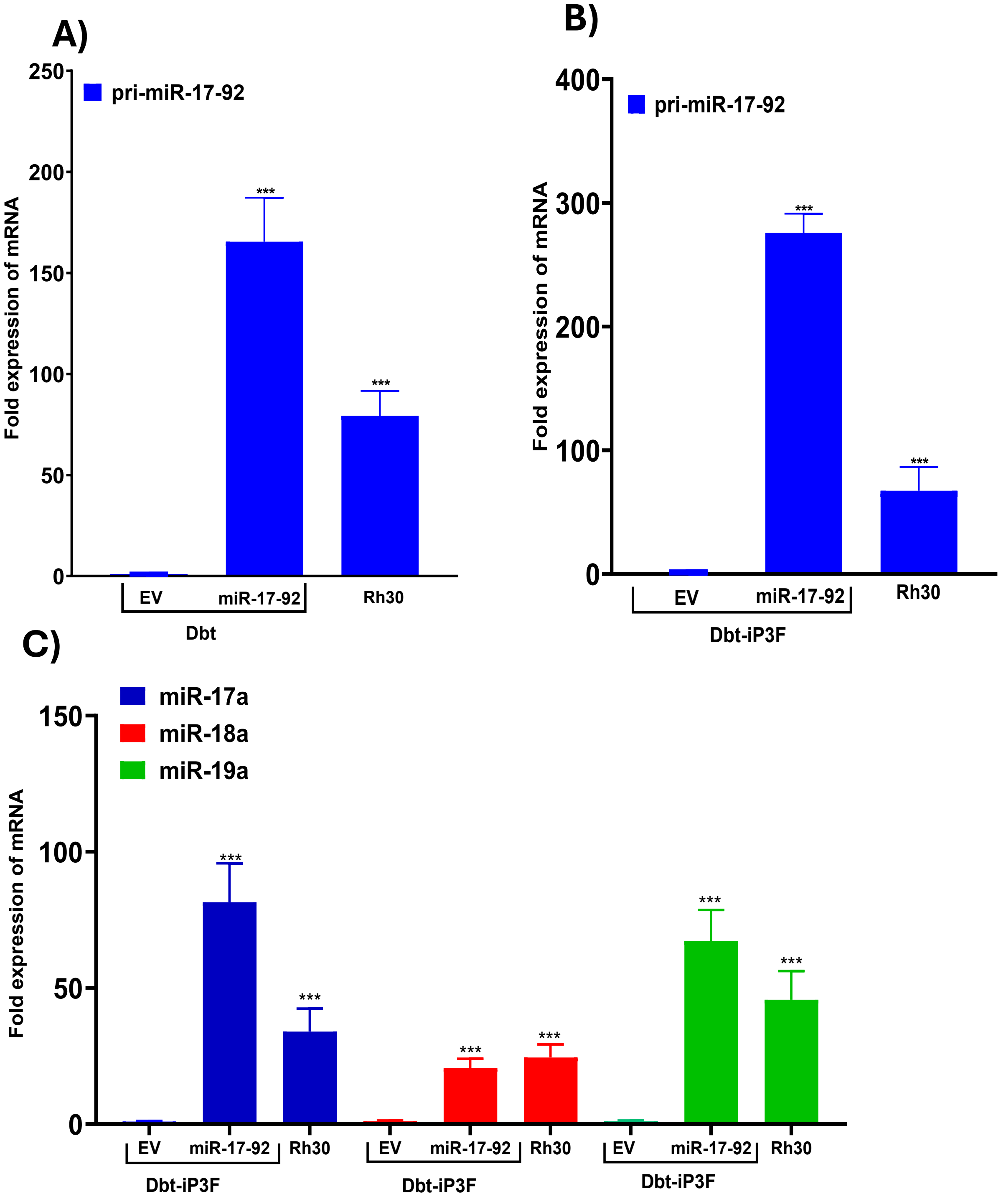

Figure S4. Effects of miR-17-92 expression in Dbt wild-type and Dbt-iP3F cells. A. qPCR analysis of pri-miR-17-92 expression in wild-type Dbt cells transduced with miR-17-92-containing construct or empty vector (EV) control. Data are normalized to 18S RNA and presented as mean ± SD from three replicates. **B, C.** qPCR analysis of pri-miR-17-92 (B) and mature miRNAs (C) encoded by the miR-17-92 cluster in Dbt-iP3F cells transduced with miR-17-92-containing construct or EV. Values are normalized to 18S and RNU6, respectively and presented as mean ± SD. Statistical significance is displayed as described in Figure S1.

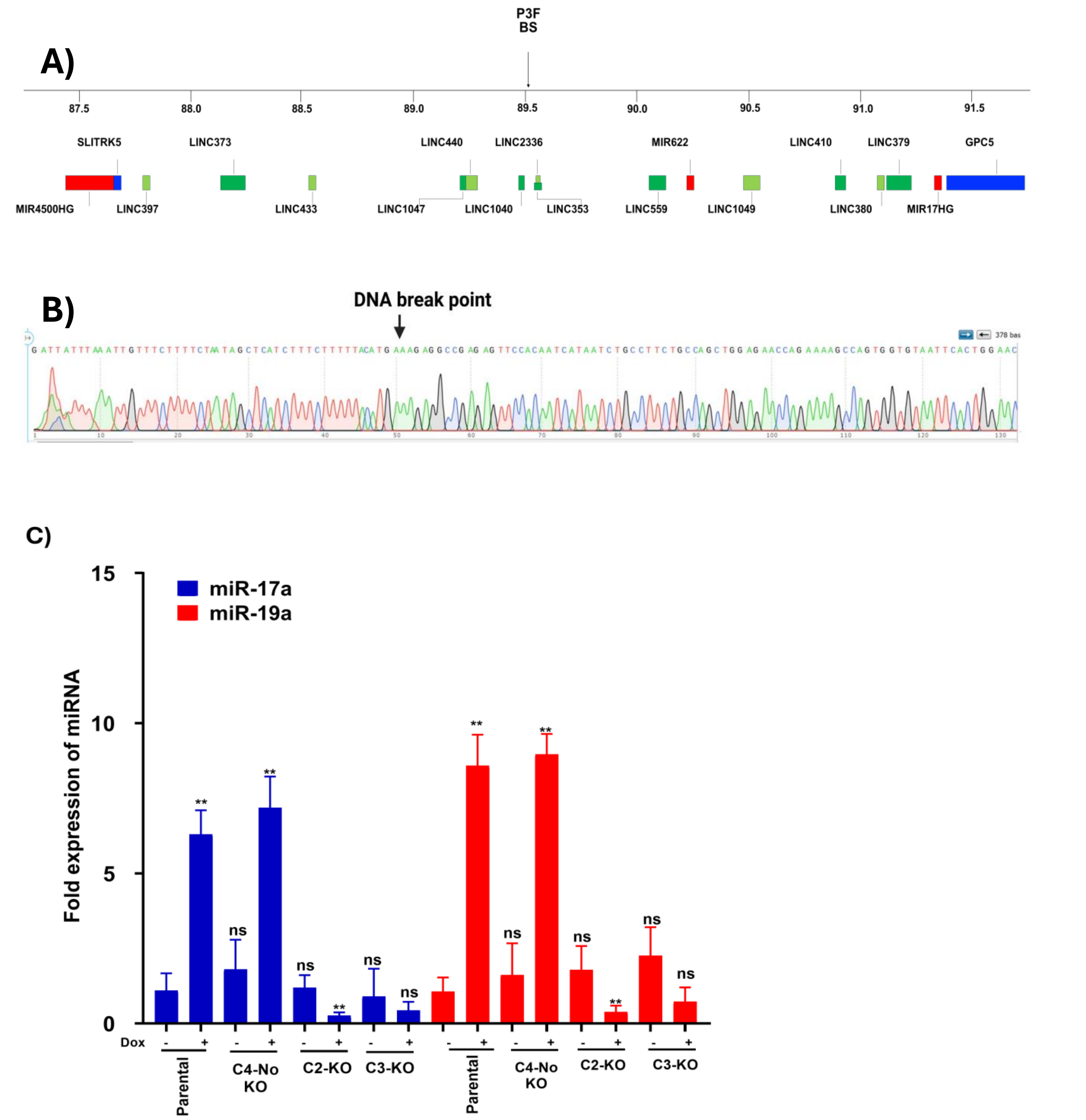

Figure S5. Identification of a distal P3F-binding site upstream of *MIR17HG*. A. Genomic map of region upstream of *MIR17HG* gene. The P3F binding site (BS) is shown above the horizontal line and distances (in Mb) are shown below the line. The horizontal boxes represent genes in this region (protein-coding, blue; miRNA-containing, red; long noncoding RNA, green). **B.** Sequencing chromatogram of deletion breakpoint in deleted subclones C2 and C3. **C.** qPCR analysis of miR-17a and miR-19a expression in deleted (C2, C3) subclones compared to parental and non-deleted (C4) controls. Cells were treated without (-) or with (+) 500 ng/ml doxycycline (Dox). Results are normalized for parental cells without Dox. Statistical significance is displayed as described in Figure S1.
